# Evaluation of a school-based dissemination of the movement guidelines for young children in Hong Kong: Study protocol

**DOI:** 10.1186/s13690-021-00705-1

**Published:** 2021-10-23

**Authors:** Catherine M. Capio, Catalina S. M. Ng, Kevin K. H. Chung, Rachel A. Jones, Cindy H. P. Sit

**Affiliations:** 1 Early Childhood Education Department, Education University of Hong Kong, Hong Kong, China; 2 School of Public Health, The University of Hong Kong, Hong Kong, China; 3 Health Science Department, Ateneo de Manila University, Quezon City, Philippines; 4 School of Education, University of Wollongong, Wollongong, Australia; 5 Department of Sports Science and Physical Education, The Chinese University of Hong Kong, Hong Kong, China

**Keywords:** Movement guidelines, Physical activity, Sedentary behavior, Sleep, Children

## Abstract

**Background:**

Healthy movement behaviors in early childhood are believed to track to adulthood, potentially imparting protective benefits against non-communicable diseases. Highlighting the collaborative and complementary roles of parents and educators in promoting health of young children, this study aims to enable parents and teachers to successfully promote healthy movement behaviors in young children. Guidelines for physical activity, sedentary screen time, and sleep of children aged 2 to 6 years will be systematically disseminated to parents and teachers of children enrolled in early childhood education centers (ECECs) in Hong Kong. An evaluation will be conducted to assess the implementation process and the outcomes of the dissemination of said guidelines.

**Methods:**

The evaluation will include formative and summative components to examine the implementation (i.e., process evaluation) and the outcomes (i.e., outcome evaluation). Participants include teachers, parents, and children from ECECs in Hong Kong. The process evaluation will be guided by the RE-AIM framework (i.e., reach, efficacy, adaptation, implementation, maintenance). Data gathering and analysis will take a mixed-methods triangulation design - convergence model. The outcome evaluation consists of a non-randomized observational study, using quantitative data from questionnaires and accelerometers. The primary outcome to be measured is the extent to which children meet the guidelines for physical activity, sedentary screen time, and sleep; the secondary outcome is teachers’ and parents’ knowledge and awareness of the guidelines.

**Discussion:**

Young children who engage in healthy movement behaviors are likely to become adults who will have the disposition to engage in behaviors that have protective effects against non-communicable diseases. The findings of this evaluation are expected to contribute to improving the strategies of systems and government agencies that aspire to promote healthy movement behaviors of young children.

## Background

In recent years, a worldwide push for promoting physical activity participation across populations has ramped up. Physical activity promotion has been shown to be of critical importance, with conclusive evidence showing physical inactivity as one of the leading risk factors for death worldwide – 3.3 million deaths per year – and a major risk factor for non-communicable diseases (NCDs) [1]. The most recent comprehensive estimate revealed that the global cost of physical inactivity to health care systems was INT$53.8 billion in the year of 2013 (INT$1 would buy in a cited country a comparable amount of goods and services that US$1 would buy in the United States) [2]. Furthermore, physical inactivity-related deaths indirectly cost INT$13.7 billion (lost productivity), and accounted for 13.4 million disability-adjusted life years (DALYs) worldwide. As such, investments to promote physical activity are warranted more than ever. Recently, the World Health Organization (WHO) released the *2020 Guidelines on physical activity and sedentary behavior* [3], which targets children aged five years and above, adolescents, adults, older adults, and specific groups that include pregnant and postpartum women, and people living with chronic conditions or disability. A year prior, the *WHO Guidelines on physical activity, sedentary behavior and sleep for children under 5 years of age* [4] was launched*.*

The guidelines for children under 5 years of age have been developed not only to mitigate physical inactivity, but also to respond to concerns related to whole-day movement behaviors (i.e., 24-h movement guidelines). From earlier work that developed similar guidelines for young children in Canada and Australia, it was established that desirable movement behaviors of young children consist not only of greater physical activity, but also of less sedentary screen time, and longer sleep [5–7]. It was further highlighted that in the early years, balanced movement behaviors gain benefits in motor and cognitive development; cardiometabolic, skeletal, and psychosocial health; and reduced risks for adiposity and injuries. These benefits exceed potential harm [8] and the movement guidelines have been gaining momentum across the globe [9]. A recent systematic review showed evidence that meeting the 24-h movement guidelines was associated with better health-related quality of life including the social-cognitive, behavioral, and emotional domains in preschool-aged children [10], indicating wider associated benefits for health.

### Movement behaviors of young children in Hong Kong

In Hong Kong, the WHO guidelines for children aged under 5 years had been adopted by the Centre for Health Protection (CHP), with the target age group modified to include 2 to 6 years old [11]. This modification reflects the age of children when they typically attend pre-primary school (i.e., early childhood education center [ECEC] which consists of nursery and kindergarten in Hong Kong). Enrollment in ECEC is not mandatory for children in Hong Kong. However, the local Education Bureau notes that virtually all children aged 3 to 5 years old attend kindergartens, based on the data recorded in the 2019/20 school year [12]. The guidelines that were adopted by the CHP are currently being disseminated through ECECs who have volunteered to join a public health campaign that promotes healthy eating and physical activity for pre-primary school-aged children in school and home settings [13]. The ongoing public health campaign has a wider focus on the physical health of young children, and implementation of the movement guidelines has yet to be systematically examined. A deliberate and strategic implementation plan and evaluation are crucially important because key information from movement guidelines for young children need to be distilled and packaged to ensure effective dissemination to stakeholders [14].

Hong Kong has a rapidly ageing population, where adults aged 65 years old and above are expected to make up as much as 33% of the population in 2039 and 38% of the population in 2069 [15]. Thus, getting children towards a path of healthy movement behaviors early is an important contributor to managing a future super-aged society. Research has shown that childhood activity patterns track to adulthood [16], and active adults in the future would contribute to mitigating the health care burden of a super-aged population. However, it appears that further work is needed to achieve this. The most recent survey by the Hong Kong Department of Health on the physical activity of pre-primary school children revealed that less than 30% achieved the recommended 180 min of daily physical activity [17]. Moreover, only 15% of parents were aware of the physical activity recommendations for children aged 2 to 6 years. In contrast, recommendations on sedentary screen time appears to be better applied as the children’s median screen time was reported to be 60 min, and 79% of parents were aware of the screen time recommendations for young children. There has been no information thus far on the sleep patterns of young children.

While the health authorities in Hong Kong have adopted the WHO-recommended movement guidelines for young children, there is currently limited knowledge of the uptake of these movement behaviors in home and school settings. There is clearly a need to promote healthy movement behaviors in young children through mechanisms that involve the adults that they interact with (e.g., parents, teachers). Thus, a systematic and deliberate approach to empower parents and teachers by enhancing their knowledge and understanding of healthy movement behaviors is crucial.

### The current study

The current strategies that are in place to promote healthy movement behaviors of young children in Hong Kong could be enhanced by leveraging the collaborative and complementary roles of parents and educators. In this current study, we aim to enable parents and teachers, so that they may successfully promote healthy movement behaviors in pre-primary school-aged children. The movement guidelines for children aged 2 to 6 years, which have been issued by the CHP will be systematically disseminated to parents and teachers of children enrolled in ECECs in Hong Kong. The dissemination plan will be informed by active engagement with stakeholders through formative work. An evaluation will be conducted to assess the implementation process and the outcomes of the dissemination. Specifically, the evaluation will: (1) assess the uptake of the dissemination of the movement guidelines by parents and teachers of children in ECECs, (2) identify the barriers and enablers to implementation of the movement guidelines in the local context, and (3) evaluate the impact of the school-based dissemination on parents, teachers, and children.

## Materials and methods

### Dissemination plan

Public health strategies will be successful if they are tailored to suit local contexts and responds to stakeholders’ needs [18]. As such, formative work was conducted to inform the design of the dissemination plan. Focus group discussions (FGDs) and interviews are established methods to effectively gain meaningful insights from, and assure involvement of, stakeholders [19, 20]. Twelve focus group discussions (FGDs) were conducted in six ECECs which involved teachers (*n* = 18) and parents (*n* = 18) of children enrolled in the respective ECECs (i.e., one FGD for each participant group in each ECEC; three participants in each FGD). Two experienced researchers facilitated the discussions which explored the participants’ (1) awareness and knowledge of the movement guidelines for young children in Hong Kong, and (2) perspectives on how these guidelines might be disseminated effectively so that adults can promote them in children. Open-ended questions were asked to initiate the discussions with participants (e.g., “what kinds of activities would help the teaching staff in your kindergarten understand the movement guidelines for children in Hong Kong?”). To mitigate the possibility of some FGD participants dominating the discussion [21], the facilitators prompted each participant to contribute any further thoughts to the discussion prior to transition of topics. Six individual interviews were conducted with stakeholder representatives (i.e., ECEC principal, ECEC program director, ECEC leadership and management expert, family psychologist, early childhood education academic, and public health academic). The interviews explored the participants’ (1) understanding of the system factors that enable and hinder healthy movement behaviors of young children in Hong Kong, and (2) perspectives on how the movement guidelines might be understood and promoted by teachers and parents in the local context.

The data gathered through the FGDs and interviews were examined using a six-phase analytic approach to thematic analysis [22]. It was determined that teachers and parents were generally aware that there are movement guidelines for young children in Hong Kong, but their knowledge of the specifications (i.e., minutes, hours), rationale, and benefits associated with these guidelines was limited. Furthermore, time constraints that affect teachers (i.e., classroom scheduling) and parents (i.e., long working hours) hinder their ability to ensure that children are meeting the movement guidelines. It was also confirmed that the socio-cultural context that currently prevails in Hong Kong puts an emphasis on early academic pursuits which are viewed as crucial to get children on a path to success. This perspective inevitably influences the learning activities in ECECs and the patterns of daily activities at home and in the wider community.

Based on the findings of the formative work, the following content will be disseminated: (1) specifications and rationale behind the movement guidelines, (2) benefits that children might have by meeting each of the guidelines, and (3) practical strategies that can be implemented daily by teachers (i.e., in class) and parents (i.e., daily activities) to promote uptake of the guidelines. Furthermore, the dissemination plan also needs to address issues that relate to: (1) teachers’ constraints due to the current curriculum expectations, (2) parents’ constraints due to competing work demands, (3) local caregiving practices such as participation of grandparents and nannies, and (4) motivation of adults to engage in healthy movement behaviors themselves.

The dissemination activities that are summarized in Table 1 will take place over 8 months of one school year (i.e. September 2021 to May 2022). Educational meetings (i.e., workshops, seminars) will be held separately for two participant groups (i.e. teachers and principals, parents and primary caregivers) in each participating ECEC. Trained project staff will conduct face-to-face sessions in the first month of the implementation; and offer subsequent sessions on a quarterly basis for reinforcement of shared ideas and concepts. These meetings will facilitate the stakeholders’ understanding of “why” (i.e. rationale) and “how” (i.e. actions) in relation to supporting children to meet the movement guidelines. A maximum of three face-to-face sessions could be conducted in each ECEC over the implementation period; the actual number would be recorded for the evaluation (see Process Evaluation section). Continuous engagement will be done through dedicated social media platforms (e.g., Facebook, WhatsApp). Informational and educational materials will be distributed to parents and teachers in multiple media (i.e. print, images, videos) over the eight-month period. Consistent with the *Kindergarten Education Curriculum Guide of Hong Kong* [23], the materials will integrate recommendations and practices with daily life activities of children in ECECs. The materials will equip the target adult recipients with a range of practicable ideas. A dedicated project staff will monitor the social media platforms, provide timely feedback, and facilitate knowledge sharing.

**Table 1:** Activities for dissemination of the movement guidelines for young children

Participant Groups	Activity	Description
(1) teachers and principals (2) parents and primary caregivers	Educational meetings (workshop/seminar)	• Conducted on-site of the ECEC and delivered separately for the participant groups. • Introduction of the movement guidelines; included are the rationale and evidence behind the guidelines, and practical strategies that can enable children to meet the guidelines. • Content tailored according to the needs of the participant groups (e.g., lesson context for teachers). • Minimum of one meeting per participant group; ECECs may request follow-up sessions (maximum of three sessions per participant group in total).
	Social media	• Reminders through online messaging chat groups (e.g., WhatsApp) will reinforce the concepts that were introduced in the educational meetings. • Participants can ask questions/clarifications, which the project team will respond to. Chat groups will be set up separately for the participant groups in each ECEC. • Practical strategies for daily activities will be shared through Facebook and/or Instagram • Minimum of bi-weekly updates will be initiated by the project team; participants may ask questions at any time.
	Resource materials	• Printed materials (i.e., posters, brochures, activity sheets) will be made available in the ECECs for teachers and parents to take. • Multi-media such as videos and picture cards will be made available online (e.g. YouTube) and will be linked to social media updates.

### Evaluation plan

The evaluation will include a formative component to examine the implementation (i.e., process evaluation) and a summative component to examine the outcomes (i.e., outcome evaluation) [24]. The formative component will enable early resolution of barriers and enhancement of facilitators. The summative component will examine the overall implementation, and identify the factors that influenced the delivery of outcomes.

#### Recruitment

Eight ECECs, from across the three major districts of Hong Kong (i.e., Hong Kong Island, Kowloon, New Territories), will be recruited to join the implementation. The inclusion criteria for ECECs are that they: (1) are included in the published list of kindergartens by the Education Bureau, (2) follow the *Kindergarten Education Curriculum Guide of Hong Kong* [23], and (3) offer classes across three kindergarten levels (i.e., K1, K2, K3). A second group of eight ECECs, who meet the same eligibility criteria and with comparable background to the first group (i.e., ECEC size, location), will also be recruited to join a comparison group for the outcome evaluation. This second group will participate in the activities for the dissemination of the movement guidelines (i.e., educational meetings, social media, resource materials) in the school year following the implementation period. Given the constraints associated with the recruitment of ECECs to participate in projects during a pandemic, the recruitment process will be purposive. The timeline for joining the project will be the prerogative of each ECEC (i.e., hence, the group to which they will be allocated to).

#### Participants and sample size

In the formative evaluation, participants will consist of teachers (*n* = 24), administrators/ principals (*n* = 8), and parents/primary caregivers of children aged 3 to 6 years (*n* = 240) from the first group of eight ECECs (i.e., the implementation group).

In the outcome evaluation, the required sample size was calculated for the planned logistic regression analysis of the primary outcome (see Data Processing and Analysis section). It was calculated that assuming an effect size of adjusted odds ratio = 1.5, alpha at 0.05, and power at 80%, a total sample size of *N* = 242 parents/primary caregivers (and their children) is required from the two groups of ECECs (i.e., the implementation and comparison groups). With an assumed 40% non-response rate from the combined implementation and comparison groups (*N* = 480 parents) and a further 10% with incomplete data, we expect complete responses from a sample size of *N* = 240.

#### Formative process evaluation

The process evaluation will be guided by the RE-AIM framework [25], which consists of five elements that are known to improve the adoption and implementation of evidence-based health interventions. These five elements include: reach (number, proportion, and extent of participation), efficacy (impact of the guidelines on the teachers, parents, and children), adoption (organizational support for the guidelines), implementation (delivery and use of the guidelines), and maintenance (long-term awareness and use of the guidelines). Table 2 summarizes the application of the RE-AIM framework in this study alongside the planned methodology.

**Table 2:** Process evaluation based on the RE-AIM framework [25] and using a mixed-methods approach

ELEMENT	METHOD	INDICATORS	EVALUATION QUESTIONS
Reach	Participation audit	Participation in educational meetings	What proportion of the target participants attended the educational meetings? How many educational meetings were conducted in each participating ECEC?
	Online metrics	Volume and frequency of activities on the online platforms	How frequent did the participants visit the online platforms (website, Facebook)? What are the materials that the participants viewed, interacted with, or downloaded (e.g. brochures, posters, videoclips)? How frequent did the participants leave comments on the available materials? Describe the comments and feedback left by participants on the online platforms.
Efficacy	Online survey	Participants’ perceptions and knowledge	How satisfied were the participants with the dissemination activities and materials? To what extent did parents and teachers know and understand the guidelines for (1) physical activity, (2) sedentary behavior, and (3) sleep?
	Focus group discussion	Supportive strategies enacted and reported by teachers and parents	Describe the strategies that teachers and parents implemented to support children in meeting the guidelines.
Adoption	Focus group discussion	Supportive strategies reported by ECEC administrators/principals	Describe the strategies that ECECs implemented to support the teachers and parents to apply their knowledge to promote healthy movement behaviors.
Implementation	Online survey	Participants’ perceptions	To what extent did the participants find the information and materials to be practicable, accessible, and useful?
	Focus group discussion	Factors related to delivery of activities and information Factors related to implementation of activities by participants	What were the factors that influenced the delivery of information? What were the factors in the school, home, community, social context, or other situational issues that influenced the children in meeting the guidelines (barriers and enablers)?
Maintenance	Online survey	Participants’ knowledge Behaviors of children as reported by teachers and parents	To what extent did parents and teachers know and understand the movement guidelines? To what extent did children in participating ECECs meet the guidelines?

A mixed-methods triangulation design – convergence model with equal weight drawn from the quantitative and qualitative components (i.e., QUAN + QUAL) will be used to gather and analyze data [26]. In this design, quantitative and qualitative data collection methods will be conducted concurrently at each data collection time point, analyzed separately, and converged upon interpretation. This design has been recommended as a means to achieving well-substantiated conclusions on the implementation process. Moreover, a mixed-methods approach to evaluation using the RE-AIM framework has been known to facilitate understanding of complex situations and the factors that lead to implementation outcomes [27]. Quantitative data will be gathered through participation audit, online metrics, and close-ended questions in online surveys. Qualitative data will be gathered through open-ended questions in online surveys and focus group discussions (FGDs).

Participation audit will consist of monitoring the number of educational meetings in each ECEC and the participants’ attendance in the said educational meetings. Online metrics will include the recording of interactions with media that are made available through the social media platforms (i.e., number of hits, likes, shares or downloads of materials), including comments that are left by participants on the available media. Online surveys will be administered at two time points: 3 months (Time 1, T1) and 6 months into the implementation (Time 2, T2).

The online surveys will assess teachers’ and parents’ satisfaction with the educational meetings, perceptions of the usefulness of materials, and knowledge and understanding of the movement guidelines. For the formative component, responses to the online survey will inform the project team so that actions to mitigate barriers and enhance facilitators can be initiated. Based on questions used in previous studies that examined the dissemination of physical activity guidelines [28–30], the online survey questions will include dichotomous questions (i.e., Yes/No), five-point Likert-type scales, and open-ended questions to assess participants’ knowledge and awareness of the movement guidelines.

FGDs will be conducted at T1 and T2 with a subsample of participants (i.e. teachers and principals, *n* = 3 per ECEC, total *n* = 24; parents or primary caregivers, *n* = 3 per ECEC, total *n* = 24) to query the strategies that teachers and parents implemented to enable children to meet the guidelines, and the strategies that ECEC principals implemented to support the teachers and parents. The target sample sizes for the FGDs exceed the recommended sample size for FGDs in projects of this current scale [31]. The discussions will also explore the factors that influenced the dissemination of information, and the factors that challenged and enabled the children meeting the guidelines [32, 33].

#### Summative outcome evaluation

A non-randomized observational study design will be used for the outcome evaluation, where outcomes will be compared between the implementation group and the comparison group. The implementation group will consist of teachers and parents from the ECECs that participated in the dissemination activities; the comparison group will consist of teachers and parents from a matched group of ECECs who have yet to join the implementation. ECECs in the comparison group will join educational meetings and receive all materials in the subsequent school year after the implementation period. The primary outcome of interest is the extent to which children in the ECEC groups meet the guidelines for physical activity, sedentary screen time, and sleep. The secondary outcomes are the knowledge and awareness of healthy movement guidelines by teachers and parents.

Teachers and parents will be invited to respond to online surveys at two additional time points: immediately following the implementation period (Time 3, T3), and 4 months post-implementation (Time 4, T4). These surveys will measure the secondary outcome by assessing the participants’ knowledge and awareness of the movement guidelines. The surveys for parents will include additional questions to measure the primary outcome by assessing the extent to which children meet the guidelines through a parent-proxy questionnaire. The said proxy-report questionnaire is made up of items from validated English-language questionnaires [34, 35] which were translated and back-translated [36] for use among parents of young children in Hong Kong. The translated questionnaire had been found to have good internal consistency (Cronbach’s alpha = 0.762), and is currently being used in an ongoing longitudinal study of child development in Hong Kong. Finally, open-ended questions will query on family strategies that relate to children meeting the movement guidelines.

For a further measure of the primary outcome, parents will also be invited to let their children wear accelerometers for objective measurement of physical activity, sedentary behavior, and sleep. The Actigraph GT3X (Actigraph, Pensacola, FL, USA) will be used, which is an accelerometer equipped with antero-posterior, vertical, and medio-lateral axes [37]. The GT3X is a widely used device for physical activity and sedentary behavior monitoring, and cut-points have been validated for young children [38]. Evidence-based accelerometer data collection for children will be followed [39]. Accelerometer epoch length will be set at 15 s, and each child will be asked to wear the GT3X above the right hip on a waist belt during waking hours for seven consecutive days. The parents/caregivers will also be instructed to remove the GT3X during water-based activities (e.g., bathing, swimming), and to record the time when the GT3X was put on and removed (i.e., record the wear/non-wear time in a diary).

The data gathering procedures for the process and outcome evaluations are summarized in Table 3, according to the timeline of measurements.

**Table 3:** Timeline of data gathering procedures for the process and outcome evaluations

	Time 1	Time 2	Time 3	Time 4
Participation audit	•	•		
Online metrics	•	•		
Online survey (knowledge and awareness of movement guidelines)	•	•	•	•
Focus group discussions	•	•		
Online survey (parent-proxy questionnaire on primary outcome)			•	•
Accelerometer monitoring			•	

#### Data processing and analysis

For the process evaluation, quantitative data obtained from the participation audit, online metrics, and online survey (close-ended questions) will be analyzed using descriptive statistics, and explored using cross-tabulations to identify differences (if any) between participant groups (i.e., teachers/administrators, parents/caregivers) and between ECECs. Qualitative data obtained from the online survey and outcomes questionnaire (open-ended questions), and transcribed FGDs will be examined using thematic analysis, guided by a realist framework which assumes that language captures participants’ experiences of reality [40]. A six-phase analytic process will be adopted consisting of: familiarizing with the data, generating codes, generating initial themes, reviewing and developing themes, refining, defining, and naming themes, and writing the report [22]. Coding and theme generation will take inductive (i.e., bottom up approach) and semantic approaches (i.e., explicit meaning of gathered data) as these are known to align well with a realist framework [40]. Thematic analysis will be supported by NVivo 12.0.

Findings from the quantitative and qualitative analyses will be converged, guided by the RE-AIM framework [25] to answer the evaluation questions (see Table 1). The converged analysis will account for participant groups and individual ECECs, accounting for differences in the background and volume of engagement during the implementation.

For the outcome evaluation, the proportions of children who meet the guidelines will be measured based on the parent-proxy questionnaire and accelerometer monitoring. A minimum of 4 days of accelerometer data will be required to be considered valid [39]; cut-points developed by Evenson et al. [41] and Pate et al. [42] have been recommended to determine time spent in physical activity and sedentary behavior in young children [37]. Meeting the guidelines is defined as ≥180 min/day of total physical activity (of which 60 min is vigorous physical activity), ≤1 h/day of sedentary screen time, and 10–13 h of sleep per 24-h period [4]. Logistic regression will be conducted to determine the contribution of the dissemination activities (i.e., implementation or comparison group), child characteristics, and ECEC on the likelihood of a child meeting each guideline component (i.e., primary outcome).

The secondary outcome of knowledge and awareness of the movement guidelines by parents and teachers will be compared between the implementation and comparison groups using descriptive and inferential statistics, whilst accounting for participant characteristics (e.g., age, sex) and ECEC. Parametric statistics will be used when assumptions of normality are met. All statistical analysis procedures will be performed on SPSS 26.0 with statistical significance set at *p* < 0.05.

### Ethical considerations

The study was approved by the Human Research Ethics Committee of the Education University of Hong Kong (reference number 2019–2020-0145). Participating ECECs will be recruited through their principals, who will provide written consent for their participation. Subsequently, teachers and parents will provide written consent prior to joining any data gathering procedure. Participants who do not consent to participate in the study may still join the educational meetings and use the social media platforms, but they do not have to participate in any of the data gathering procedures. The potential benefits of the dissemination plan will not be withheld from ECECs in the comparison group because they will receive the same dissemination activities following completion of the outcome evaluation. All participants will also be invited to provide written consent for their identifiable data to be held by the research team for possible future follow-up for no longer than 5 years following study completion.

The findings of this study will be disseminated in scientific conferences and peer-reviewed journals within the areas of public health and child development. Knowledge will also be shared with teachers and parents through continuous social media platforms to support more teachers and parents in enabling young children to meet the movement guidelines. Finally, insights from the implementation will be shared with the Centre for Health Protection of Hong Kong, to inform further health promotion efforts by relevant government agencies.

### Status and timeline of the study

The study implementation commenced in September 2021. Based on the planned timeline, the implementation will continue until May 2022, and T4 data gathering is expected in September 2022.

## Discussion

The project aims to contribute towards improved health behaviors of Hong Kong young children through the enabling actions of the adults who support their development. It is believed that by targeting early childhood, the work would potentially contribute to long-term health promotion and prevention of non-communicable diseases (NCDs) as movement behaviors are known to track from childhood to adulthood [15]. Young children who engage in healthy movement behaviors are likely to become adults who will have the disposition to engage in behaviors that have protective effects against NCDs. Considering the costs that physical inactivity and highly sedentary behavior imposes on the health care system, promoting healthy movement behaviors early in childhood could contribute towards sustainable health care services. While it is outside the scope of this current project, a follow-up study could be designed to quantify the costs and benefits of investing in early childhood through actuarial models that measure the social return on investment [43].

In the short-term, the outcomes of this study directly contribute to improving the strategies of the government agencies in Hong Kong that are tasked to promote health and development among young children (i.e., health, education). The movement guidelines for pre-primary school-aged children are based on evidence that had been evaluated through robust processes [4]. By leveraging on established evidence while accounting for the local context, this project contributes to health promotion through sensible and economical utilization of resources.

### Potential challenges and mitigations

Due to the duration of the project, challenges are bound to occur. It is assumed that stakeholders would continue to be engaged over the implementation period. However, there is a risk that in the eight-month period, teachers and parents would become disengaged. To mitigate this, project staff overseeing the social media channels will ensure a continuous flow of information and feedback with the participants.

It is also acknowledged that parents in Hong Kong are known to have a bias that prioritizes academic pursuits in promoting child development [44]. Because Hong Kong parents are known to be in a position to encourage or discourage physical activity participation [45], this project will need to ensure that parents are convinced of the importance of healthy movement behaviors and the positive associations between healthy movement behaviors and academic outcomes are reiterated. Findings from the formative study which informed the dissemination plan will ensure that parents’ bias will be managed. Finally, objective (i.e., accelerometers) and subjective (i.e., questionnaire) measures of movement behaviors each have associated challenges that could impact the veracity of gathered data; the use of both approaches is meant to make up for the limitations of using them in isolation.

With the challenges mitigated, this study stands to generate findings that will inform subsequent public health promotion programs for young children in Hong Kong. The evaluation will deliver information on the uptake of the movement guidelines, the barriers and enablers that can aid uptake, and the impact of deliberately engaging teachers and parents in the promotion of healthy movement behaviors of young children. The lack of randomization for the outcome evaluation may pose as a limitation, but the combination with process evaluation will likely generate meaningful insights that will inform future implementations. While the study is contextualized to Hong Kong, the findings could be useful to other territories’ efforts of disseminating movement guidelines in young children.

## Data Availability

Data will be saved and made available subsequently through the following OSF registration: Capio CM, Chung KKH, Ng CSM, Jones RA, Sit CHP. 2021. Implementation Science Approach to Disseminating Healthy Movement Guidelines for Young Children in Hong Kong. OSF. osf.io/mfut9.

## Abbreviations

CHP: Centre for Health Protection (Hong Kong)
DALY: Disability-adjusted life years
ECEC: Early childhood and education center
FGD: Focus-group discussion
NCD: Non-communicable diseases
RE-AIM: Reach, efficacy, adoption, implementation maintenance
WHO: World Health Organisation
